# Crystal structures of 2-(2-bromo-5-fluoro­phen­yl)-8-eth­oxy-3-nitro-2*H*-thio­chromene and 2-(2-bromo-5-fluoro­phen­yl)-7-meth­oxy-3-nitro-2*H*-thio­chromene

**DOI:** 10.1107/S2056989019014178

**Published:** 2019-10-31

**Authors:** Chien Thang Pham, Dinh Hung Mac, Thai Thanh Thu Bui

**Affiliations:** a Faculty of Chemistry, VNU University of Science, Vietnam National University, Hanoi, 19 Le Thanh Tong, Ha Noi, Vietnam

**Keywords:** crystal structure, thio­chromene, hydrogen bonding, π–π stacking

## Abstract

Two thio­chromene com­pounds containing Br and F atoms, namely 2-(2-bromo-5-fluoro­phen­yl)-8-eth­oxy-3-nitro-2*H*-thio­chromene (C_17_H_13_BrFNO_3_S, **A**) and 2-(2-bromo-5-fluoro­phen­yl)-7-meth­oxy-3-nitro-2*H*-thio­chromene (C_16_H_11_BrFNO_3_S, **B**), were prepared *via* the condensation reaction between 2-mer­capto­benzaldehyde and nitro­styrene derivatives. In both com­pounds, the thio­chromene plane is almost perpendicular to the phenyl ring. In the structure of **A**, mol­ecules are assembled *via* π–π stacking and C—H⋯O and C—F⋯π inter­actions. In the crystal packing of **B**, mol­ecules are linked by C—H⋯F, C—H⋯O, C—H⋯π and π–π inter­actions.

## Abstract 2

The synthesis and structure of 2-(2-bromo-5-fluoro­phen­yl)-8-eth­oxy-3-nitro-2*H*-thio­chromene (**A**) and 2-(2-bromo-5-fluoro­phen­yl)-7-meth­oxy-3-nitro-2*H*-thio­chromene (**B**) are described. In each crystal, the mol­ecules are linked by hydrogen bonds and π–π stacking inter­actions.

## Chemical context   

2*H*-Chromenes (or 2*H*-benzo­pyrans) are heterocyclic com­pounds found in many natural plants. This class of mol­ecules shows a wide variety of biological activities, such as anti­cancer, anti-inflammation and anti-HIV (Horton *et al.*, 2003 ▸). Recently, we have shown that 3-nitro-2*H*-chromene can act as a selective mTOR/Pi3K inhibitor, which can lead to a new com­pound to treat breast cancer (Fouqué *et al.*, 2015 ▸). Inter­estingly, we observed that thio­chromene derivatives, where the O atom is replaced by an S atom, can increase significantly the biological activity of these com­pounds. With the goal in mind to synthesize a chemical library of thio­chromene com­pounds (Nguyen *et al.*, 2016 ▸), we have now successfully prepared 2-(2-bromo-5-fluoro­phen­yl)-8-eth­oxy-3-nitro-2*H*-thio­chromene (**A**) and 2-(2-bromo-5-fluoro­phen­yl)-7-meth­oxy-3-nitro-2*H*-thio­chromene (**B**). Crystal structure determination can help to understand the role of halogenated substituents in the biological activity of these com­pounds.
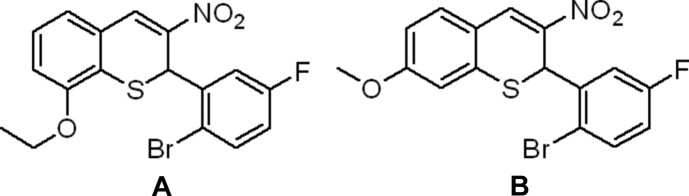


## Structural commentary   

**Figure 1:**
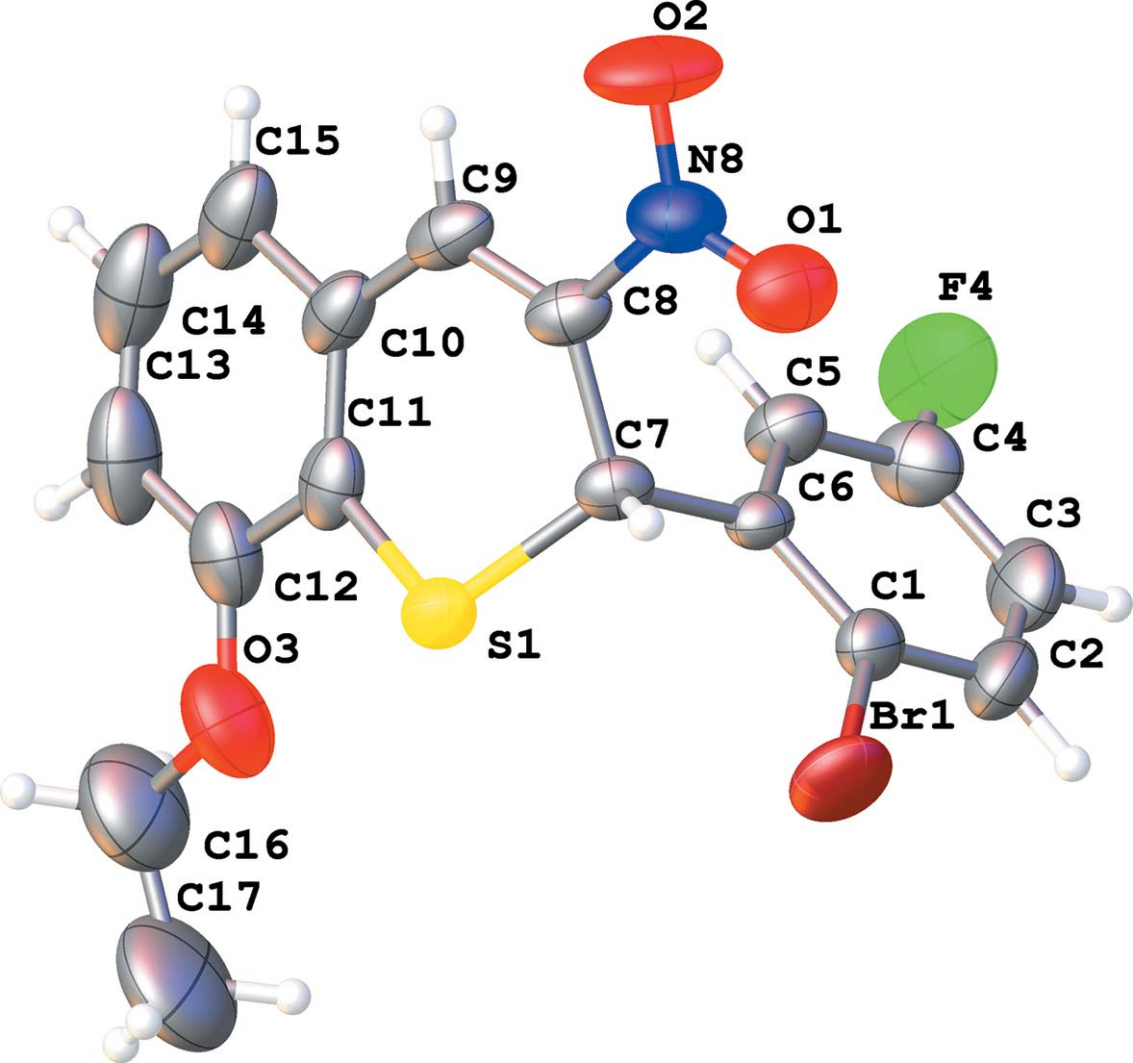
The mol­ecular structure of 2-(2-bromo-5-fluoro­phen­yl)-8-eth­oxy-3-nitro-2*H*-thio­chromene (**A**), showing the atom labeling. Displacement ellipsoids are drawn at the 50% probability level.

Compound (**A**) crystallizes in the triclinic space group *P* 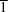, while com­pound (**B**) crystallizes in the space group *P*2_1_/*c*, both with one mol­ecule in the asymmetric unit (Figs. 1 ▸ and 2 ▸). In both com­pounds, the conformation of the thio­chromene ring is similar. In **A**, the thio­chromene ring makes an angle of 89.3 (2)° with phenyl ring C1–C6, while in **B**, this angle is 86.94 (8)°, which indicates that the 2-bromo-5-fluoro­phenyl ring is roughly perpendicular to the thio­chromene plane. Both 2*H*-thio­pyran rings have a screw-boat conformation, with atom C7 having the largest deviation from the best plane through atoms S1/C7–C11 [puckering parameters *Q* = 0.388 (4) Å, θ = 119.6 (7)° and φ = 202.2 (9)° for **A**, and *Q* = 0.5111 (18) Å, θ = 118.2 (2)° and φ = 208.1 (3)° for **B**]. The C—S bond lengths are almost equal [C7—S1 = 1.828 (4) Å and C11—S1 = 1.8307 (19) Å for **A**, and 1.758 (5) and 1.7574 (19) Å for **B**, respectively]. The C11—S1—C7 bond angle is 102.5 (2)° in **A** and 100.47 (9)° in **B**. The N—O bond lengths in com­pound **B** [1.232 (2) and 1.221 (2) Å] are slightly longer than those in com­pound **A** [both 1.219 (5) Å]. The nitro group is situated in the thio­chromene plane, as illustrated by the torsion angle O2—N8—C8—C9 of 1.3 (7)° in **A** and 9.5 (3)° in **B**.

## Supra­molecular features   

**Figure 3:**
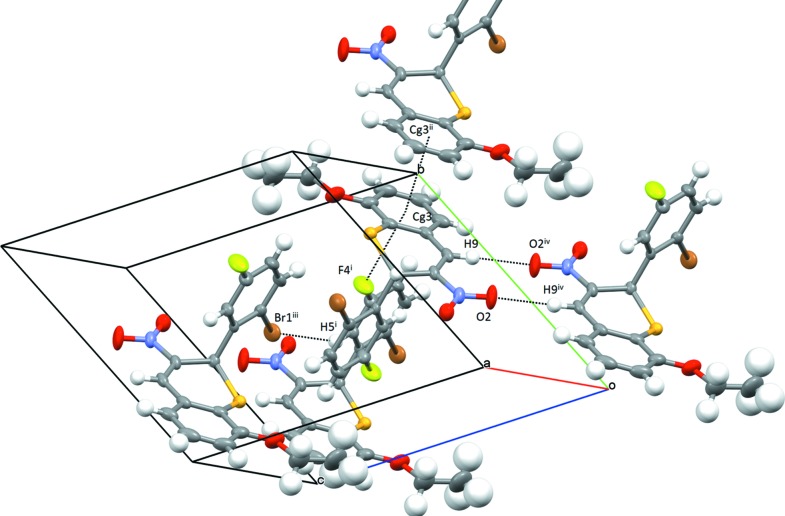
Packing diagram for **A**, showing C—H⋯O, C—F⋯π, π–π and H⋯Br inter­actions [symmetry codes: (i) −*x*, −*y* + 1, −*z* + 1; (ii) −*x*, −*y* + 2, −*z*; (iii) −*x* + 1, −*y* + 1, −*z* + 1; (iv) −*x*, −*y* + 1, −*z*]. *Cg*3 is the centroid of the C10–C15 ring.

In the crystal of **A**, mol­ecules form inversion dimers *via* C—H⋯O hydrogen bonds (Table 1 ▸ and Fig. 3 ▸) and π–π inter­actions [*Cg*3⋯*Cg*3^i^ = 3.646 (3) Å; symmetry code: (i) −*x*, −*y* + 2, −*z*; *Cg*3 is the centroid of the C10–C15 ring]. Neighbouring dimers inter­act through C—F⋯π and short Br1⋯H5^ii^ inter­actions [F4⋯*Cg*3^ii^ = 3.328 (4) Å and Br1⋯H5^iii^ = 2.96 Å; symmetry codes: (ii) −*x*, −*y* + 1, −*z* + 1; (iii) *x* + 1, *y*, *z*].

**Figure 4:**
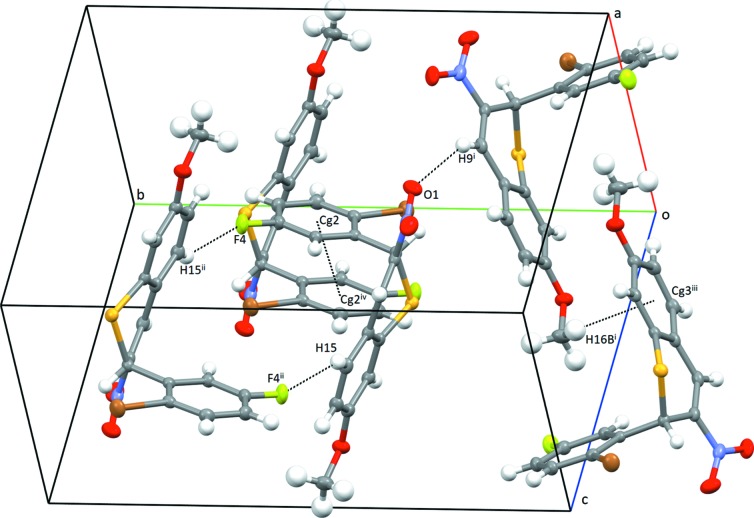
Packing diagram for **B**, showing C—H⋯O, C—H⋯F, C—H⋯π and π–π inter­actions [symmetry codes: (i) *x*, −*y* + 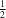, *z* − 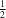; (ii) −*x* + 2, −*y* + 1, −*z* + 2; (iii) −*x* + 1, *y* − 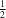, −*z* + 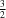; (iv) −*x* + 1, −*y* + 1, −*z* + 1]. *Cg*2 and *Cg*3 are the centroids of the C1–C6 and C10–C15 rings, respectively.

In the crystal of com­pound **B**, two mol­ecules form dimers through C—H⋯F hydrogen bonds (Table 2 ▸ and Fig. 4 ▸). These dimers form chains running in the *c* direction through π–π inter­actions [*Cg*2⋯*Cg*2^i^ = 3.8458 (13) Å; symmetry code: (i) −*x* + 2, −*y* + 1, −*z* + 1; *Cg*2 is the centroid of the C1–C6 ring]. Parallel chains inter­act *via* C—H⋯O and C—H⋯π inter­actions.

## Database survey   

The Cambridge Structural Database (CSD, Version 5.40, update of May 2019; Groom *et al.*, 2016 ▸) contains seven phenyl-2*H*-thio­chromene derivatives, of which three contain halogen atoms [CSD refcodes IFOZIO (Choudhury & Mukherjee, 2013 ▸), QAPSAE (Simlandy & Mukherjee, 2017 ▸) and WAPCUO (Sangeetha & Sekar, 2017 ▸)] and only one structure contains a nitro substituent on the 2*H*-thio­chromene ring (NOGDIZ; Le *et al.*, 2019 ▸). In all seven structures, the phenyl ring is roughly perpendicular to the thio­chromene plane, with dihedral angles between 87.73 and 98.89°. Four of the seven structures display inter­molecular inter­actions between the S atom and a C—H bond. However, in the two structures presented here, this type of inter­action has not been observed.

## Synthesis and crystallization   

To a round-bottomed flask was added 2-mercaptobenzaldehyde (1 equiv.), nitro­styrene (1 equiv.) and K_2_CO_3_ (1 equiv.) in toluene and the reaction mixture was stirred at room temperature for 2 h. After com­pletion of the reaction, the solvent was evaporated under reduced pressure and the crude product was purified by flash chromatography on silica gel (yield 90%). Crystals suitable for single-crystal X-ray diffraction data collection were obtained by slow evaporation from an ethanol solution.

## Refinement   

Crystal data, data collection and structure refinement details are summarized in Table 3 ▸. All H atoms bonded to C atoms were placed at calculated positions, with C—H = 0.93–0.98 Å, and refined as riding, with *U*_iso_(H) = 1.2*U*_eq_(C) for C*sp* ^2^—H and *U*_iso_(H) = 1.5*U*_eq_(C) for C*sp* ^3^—H. A rotating-group model was applied for methyl-group C17 in **A** and C16 in **B**.

## Supplementary Material

Crystal structure: contains datablock(s) A, B, global. DOI: 10.1107/S2056989019014178/vm2223sup1.cif

Structure factors: contains datablock(s) A. DOI: 10.1107/S2056989019014178/vm2223Asup8.hkl

Structure factors: contains datablock(s) B. DOI: 10.1107/S2056989019014178/vm2223Bsup9.hkl

Supporting information file. DOI: 10.1107/S2056989019014178/vm2223Asup4.cml

Supporting information file. DOI: 10.1107/S2056989019014178/vm2223Bsup5.cml

CCDC references: 1950406, 1953121, 1950406, 1953121

Additional supporting information:  crystallographic information; 3D view; checkCIF report

## Figures and Tables

**Figure 2:**
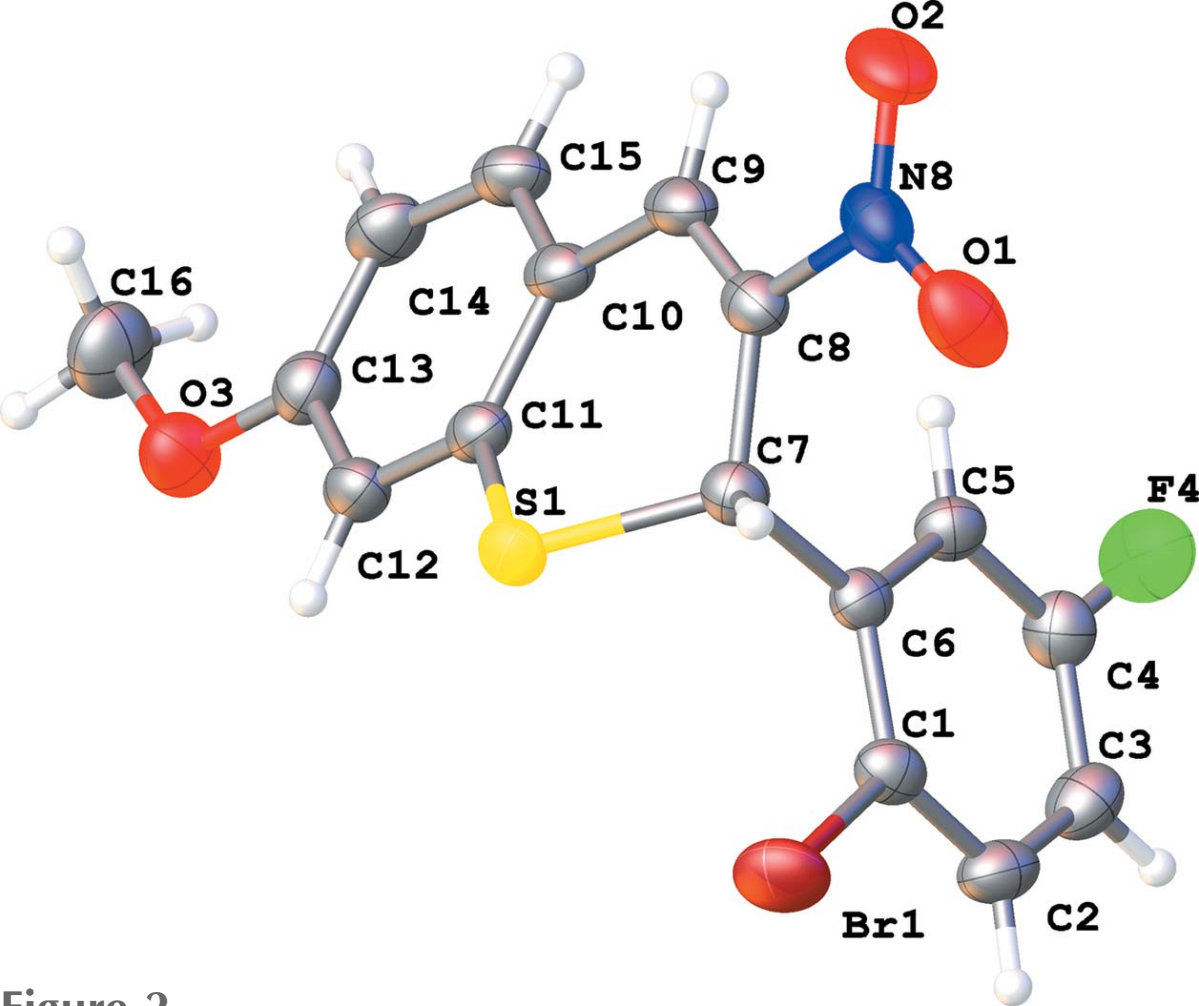
The mol­ecular structure of 2-(2-bromo-5-fluoro­phen­yl)-7-meth­oxy-3-nitro-2*H*-thio­chromene (**B**), showing the atom labeling. Displacement ellipsoids are drawn at the 50% probability level.

**Table 1:** Hydrogen-bond geometry (Å, °) for **A** [Chem scheme1]

*D*—H⋯*A*	*D*—H	H⋯*A*	*D*⋯*A*	*D*—H⋯*A*
C9—H9⋯O2^i^	0.93	2.50	3.419 (7)	168

Symmetry code: (i) 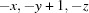.

**Table 2:** Hydrogen-bond geometry (Å, °) for **B** [Chem scheme1] *Cg*3 is the centroid of the C10–C15 ring.

*D*—H⋯*A*	*D*—H	H⋯*A*	*D*⋯*A*	*D*—H⋯*A*
C9—H9⋯O1^i^	0.93	2.60	3.418 (3)	148
C15—H15⋯F4^ii^	0.93	2.54	3.268 (2)	136
C16—C16B⋯*Cg*3^iii^	0.96	2.86	3.668 (3)	143

Symmetry codes: (i) 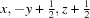; (ii) 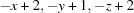; (iii) 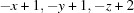.

**Table 3:** Experimental details

	**A**	**B**
Crystal data		
Chemical formula	C_17_H_13_BrFNO_3_S	C_16_H_11_BrFNO_3_S
*M*_r_	410.25	396.23
Crystal system, space group	Triclinic, *P* 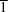	Monoclinic, *P*2_1_/*c*
Temperature (K)	273	273
*a*, *b*, *c* (Å)	7.6695 (12), 10.6867 (18), 12.2767 (19)	7.6231 (4), 17.3484 (8), 11.8345 (6)
α, β, γ (°)	64.686 (4), 80.760 (4), 70.395 (4)	90, 106.016 (2), 90
*V* (Å^3^)	856.7 (2)	1504.35 (13)
*Z*	2	4
Radiation type	Mo *K*α	Mo *K*α
μ (mm^−1^)	2.55	2.90
Crystal size (mm)	0.25 × 0.2 × 0.15	0.30 × 0.22 × 0.11

Data collection		
Diffractometer	Bruker D8 Quest CMOS	Bruker D8 Quest CMOS
Absorption correction	Multi-scan (*SADABS*; Bruker, 2016 ▸)	Multi-scan (*SADABS*; Bruker, 2016 ▸)
*T*_min_, *T*_max_	0.621, 0.745	0.599, 0.746
No. of measured, independent and observed [*I* > 2σ(*I*)] reflections	12684, 3255, 2291	31660, 3745, 3018
*R*_int_	0.037	0.032
(sin θ/λ)_max_ (Å^−1^)	0.611	0.669

Refinement		
*R*[*F* ^2^ > 2σ(*F* ^2^)], *wR*(*F* ^2^), *S*	0.052, 0.128, 1.04	0.029, 0.070, 1.04
No. of reflections	3255	3745
No. of parameters	218	209
H-atom treatment	H-atom parameters constrained	H-atom parameters constrained
Δρ_max_, Δρ_min_ (e Å^−3^)	0.53, −0.49	0.52, −0.49

Computer programs: *APEX2* (Bruker, 2013 ▸), *SAINT* (Bruker, 2013 ▸), *SHELXT* (Sheldrick, 2015*a* ▸), *SHELXL2014* (Sheldrick, 2015*b* ▸) and *OLEX2* (Dolomanov *et al.*, 2009 ▸).

## supplementary crystallographic information

### 2-(2-Bromo-5-fluorophenyl)-8-ethoxy-3-nitro-2H-thiochromene (A) Crystal data

**Table:**

C_17_H_13_BrFNO_3_S	*Z* = 2
*M**_r_* = 410.25	*F*(000) = 412
Triclinic, *P*1	*D*_x_ = 1.590 Mg m^−^^3^
*a* = 7.6695 (12) Å	Mo *K*α radiation, λ = 0.71073 Å
*b* = 10.6867 (18) Å	Cell parameters from 5933 reflections
*c* = 12.2767 (19) Å	θ = 3.0–25.6°
α = 64.686 (4)°	µ = 2.55 mm^−^^1^
β = 80.760 (4)°	*T* = 273 K
γ = 70.395 (4)°	Triangular-prism, clear light yellow
*V* = 856.7 (2) Å^3^	0.25 × 0.2 × 0.15 mm

### 2-(2-Bromo-5-fluorophenyl)-8-ethoxy-3-nitro-2H-thiochromene (A) Data collection

**Table:**

Bruker D8 Quest CMOS diffractometer	2291 reflections with *I* > 2σ(*I*)
φ and ω scans	*R*_int_ = 0.037
Absorption correction: multi-scan (SADABS; Bruker, 2016)	θ_max_ = 25.7°, θ_min_ = 3.0°
*T*_min_ = 0.621, *T*_max_ = 0.745	*h* = −9→9
12684 measured reflections	*k* = −13→13
3255 independent reflections	*l* = −14→14

### 2-(2-Bromo-5-fluorophenyl)-8-ethoxy-3-nitro-2H-thiochromene (A) Refinement

**Table:**

Refinement on *F*^2^	Primary atom site location: dual
Least-squares matrix: full	Hydrogen site location: inferred from neighbouring sites
*R*[*F*^2^ > 2σ(*F*^2^)] = 0.052	H-atom parameters constrained
*wR*(*F*^2^) = 0.128	*w* = 1/[σ^2^(*F*_o_^2^) + (0.0419*P*)^2^ + 2.0237*P*] where *P* = (*F*_o_^2^ + 2*F*_c_^2^)/3
*S* = 1.04	(Δ/σ)_max_ < 0.001
3255 reflections	Δρ_max_ = 0.53 e Å^−^^3^
218 parameters	Δρ_min_ = −0.49 e Å^−^^3^
0 restraints	

### 2-(2-Bromo-5-fluorophenyl)-8-ethoxy-3-nitro-2H-thiochromene (A) Special details

**Table:**

Geometry. All esds (except the esd in the dihedral angle between two l.s. planes) are estimated using the full covariance matrix. The cell esds are taken into account individually in the estimation of esds in distances, angles and torsion angles; correlations between esds in cell parameters are only used when they are defined by crystal symmetry. An approximate (isotropic) treatment of cell esds is used for estimating esds involving l.s. planes.

### 2-(2-Bromo-5-fluorophenyl)-8-ethoxy-3-nitro-2H-thiochromene (A) Fractional atomic coordinates and isotropic or equivalent isotropic displacement parameters (Å^2^)

**Table:**

	*x*	*y*	*z*	*U*_iso_*/*U*_eq_	
Br1	0.78831 (7)	0.45042 (7)	0.29672 (6)	0.0676 (2)	
S1	0.34737 (16)	0.76031 (13)	0.17001 (11)	0.0459 (3)	
F4	0.1093 (5)	0.3152 (4)	0.5616 (3)	0.0878 (11)	
O1	0.4800 (5)	0.3584 (4)	0.1158 (3)	0.0627 (10)	
N8	0.3239 (6)	0.4388 (5)	0.0863 (4)	0.0514 (10)	
O3	0.1347 (6)	0.9891 (4)	0.2321 (4)	0.0800 (12)	
C6	0.4041 (5)	0.4707 (4)	0.3176 (4)	0.0317 (9)	
C7	0.3916 (5)	0.5738 (5)	0.1873 (4)	0.0344 (10)	
H7	0.5137	0.5464	0.1505	0.041*	
O2	0.2238 (6)	0.4176 (5)	0.0326 (4)	0.0877 (14)	
C1	0.5698 (6)	0.4073 (5)	0.3787 (4)	0.0398 (10)	
C5	0.2496 (6)	0.4375 (5)	0.3831 (4)	0.0430 (11)	
H5	0.1353	0.4785	0.3463	0.052*	
C8	0.2567 (6)	0.5648 (5)	0.1183 (4)	0.0394 (11)	
C9	0.0857 (6)	0.6514 (5)	0.0895 (4)	0.0441 (11)	
H9	0.0162	0.6300	0.0480	0.053*	
C10	0.0005 (6)	0.7756 (5)	0.1180 (4)	0.0449 (12)	
C11	0.1047 (6)	0.8283 (5)	0.1624 (4)	0.0440 (11)	
C2	0.5820 (7)	0.3155 (5)	0.4989 (5)	0.0540 (13)	
H2	0.6945	0.2760	0.5376	0.065*	
C4	0.2649 (7)	0.3447 (6)	0.5014 (4)	0.0538 (13)	
C12	0.0194 (8)	0.9462 (6)	0.1916 (5)	0.0588 (14)	
C3	0.4268 (8)	0.2827 (6)	0.5611 (5)	0.0593 (14)	
H3	0.4323	0.2196	0.6420	0.071*	
C15	−0.1906 (7)	0.8446 (6)	0.1038 (5)	0.0612 (15)	
H15	−0.2617	0.8100	0.0751	0.073*	
C13	−0.1723 (9)	1.0141 (6)	0.1749 (6)	0.0758 (19)	
H13	−0.2306	1.0946	0.1932	0.091*	
C14	−0.2721 (8)	0.9611 (7)	0.1315 (5)	0.0757 (19)	
H14	−0.3987	1.0062	0.1209	0.091*	
C16	0.0603 (12)	1.1016 (8)	0.2740 (8)	0.108 (3)	
H16A	−0.0355	1.0799	0.3348	0.129*	
H16B	0.0066	1.1929	0.2080	0.129*	
C17	0.2145 (15)	1.1110 (9)	0.3261 (10)	0.140 (4)	
H17A	0.3043	1.1391	0.2639	0.211*	
H17B	0.2717	1.0182	0.3877	0.211*	
H17C	0.1673	1.1819	0.3604	0.211*	

### 2-(2-Bromo-5-fluorophenyl)-8-ethoxy-3-nitro-2H-thiochromene (A) Atomic displacement parameters (Å^2^)

**Table:**

	*U*^11^	*U*^22^	*U*^33^	*U*^12^	*U*^13^	*U*^23^
Br1	0.0326 (3)	0.0840 (5)	0.0826 (4)	−0.0170 (3)	−0.0096 (2)	−0.0277 (3)
S1	0.0434 (6)	0.0424 (7)	0.0506 (7)	−0.0164 (5)	−0.0098 (5)	−0.0116 (6)
F4	0.084 (2)	0.116 (3)	0.0506 (19)	−0.054 (2)	0.0178 (17)	−0.0102 (19)
O1	0.056 (2)	0.070 (3)	0.071 (3)	−0.014 (2)	−0.0018 (19)	−0.040 (2)
N8	0.057 (3)	0.069 (3)	0.045 (2)	−0.029 (2)	0.004 (2)	−0.031 (2)
O3	0.094 (3)	0.049 (2)	0.096 (3)	−0.013 (2)	0.003 (2)	−0.036 (2)
C6	0.031 (2)	0.035 (2)	0.034 (2)	−0.0095 (18)	−0.0047 (17)	−0.0171 (19)
C7	0.029 (2)	0.046 (3)	0.034 (2)	−0.0144 (19)	0.0037 (17)	−0.020 (2)
O2	0.078 (3)	0.129 (4)	0.105 (3)	−0.037 (3)	−0.012 (2)	−0.083 (3)
C1	0.038 (2)	0.034 (2)	0.047 (3)	−0.005 (2)	−0.007 (2)	−0.018 (2)
C5	0.038 (2)	0.052 (3)	0.040 (3)	−0.016 (2)	−0.002 (2)	−0.017 (2)
C8	0.037 (2)	0.058 (3)	0.029 (2)	−0.022 (2)	0.0029 (18)	−0.019 (2)
C9	0.039 (2)	0.064 (3)	0.030 (2)	−0.026 (2)	−0.0020 (19)	−0.011 (2)
C10	0.035 (2)	0.051 (3)	0.034 (3)	−0.013 (2)	−0.0046 (19)	−0.002 (2)
C11	0.043 (3)	0.039 (3)	0.031 (2)	−0.008 (2)	0.0030 (19)	−0.002 (2)
C2	0.058 (3)	0.046 (3)	0.050 (3)	−0.004 (3)	−0.023 (3)	−0.013 (2)
C4	0.064 (3)	0.062 (3)	0.040 (3)	−0.030 (3)	0.007 (2)	−0.018 (3)
C12	0.067 (4)	0.039 (3)	0.054 (3)	−0.013 (3)	0.005 (3)	−0.008 (3)
C3	0.083 (4)	0.051 (3)	0.035 (3)	−0.021 (3)	−0.012 (3)	−0.006 (2)
C15	0.043 (3)	0.061 (4)	0.050 (3)	−0.011 (3)	−0.003 (2)	0.002 (3)
C13	0.069 (4)	0.046 (3)	0.069 (4)	0.005 (3)	0.019 (3)	−0.007 (3)
C14	0.048 (3)	0.067 (4)	0.062 (4)	−0.003 (3)	0.006 (3)	0.006 (3)
C16	0.130 (7)	0.070 (5)	0.128 (7)	−0.011 (5)	−0.009 (5)	−0.057 (5)
C17	0.187 (10)	0.072 (5)	0.179 (10)	−0.016 (6)	−0.027 (8)	−0.075 (6)

### 2-(2-Bromo-5-fluorophenyl)-8-ethoxy-3-nitro-2H-thiochromene (A) Geometric parameters (Å, º)

**Table:**

Br1—C1	1.901 (4)	C10—C11	1.400 (7)
S1—C7	1.828 (4)	C10—C15	1.403 (7)
S1—C11	1.758 (5)	C11—C12	1.379 (7)
F4—C4	1.356 (6)	C2—H2	0.9300
O1—N8	1.219 (5)	C2—C3	1.372 (7)
N8—O2	1.219 (5)	C4—C3	1.357 (7)
N8—C8	1.468 (6)	C12—C13	1.408 (8)
O3—C12	1.360 (7)	C3—H3	0.9300
O3—C16	1.418 (8)	C15—H15	0.9300
C6—C7	1.501 (6)	C15—C14	1.353 (9)
C6—C1	1.389 (6)	C13—H13	0.9300
C6—C5	1.381 (6)	C13—C14	1.367 (9)
C7—H7	0.9800	C14—H14	0.9300
C7—C8	1.488 (6)	C16—H16A	0.9700
C1—C2	1.376 (7)	C16—H16B	0.9700
C5—H5	0.9300	C16—C17	1.481 (11)
C5—C4	1.361 (6)	C17—H17A	0.9600
C8—C9	1.325 (6)	C17—H17B	0.9600
C9—H9	0.9300	C17—H17C	0.9600
C9—C10	1.433 (7)		
			
C11—S1—C7	102.5 (2)	C3—C2—C1	119.4 (5)
O1—N8—C8	117.8 (4)	C3—C2—H2	120.3
O2—N8—O1	122.5 (5)	F4—C4—C5	117.7 (5)
O2—N8—C8	119.8 (4)	F4—C4—C3	119.2 (5)
C12—O3—C16	119.7 (5)	C3—C4—C5	123.1 (5)
C1—C6—C7	121.8 (4)	O3—C12—C11	114.8 (5)
C5—C6—C7	121.3 (4)	O3—C12—C13	125.5 (6)
C5—C6—C1	117.0 (4)	C11—C12—C13	119.6 (6)
S1—C7—H7	106.6	C2—C3—H3	120.9
C6—C7—S1	111.5 (3)	C4—C3—C2	118.2 (5)
C6—C7—H7	106.6	C4—C3—H3	120.9
C8—C7—S1	111.0 (3)	C10—C15—H15	119.8
C8—C7—C6	114.2 (3)	C14—C15—C10	120.4 (6)
C8—C7—H7	106.6	C14—C15—H15	119.8
C6—C1—Br1	119.9 (3)	C12—C13—H13	120.2
C2—C1—Br1	117.9 (3)	C14—C13—C12	119.6 (6)
C2—C1—C6	122.3 (4)	C14—C13—H13	120.2
C6—C5—H5	120.0	C15—C14—C13	121.4 (6)
C4—C5—C6	119.9 (4)	C15—C14—H14	119.3
C4—C5—H5	120.0	C13—C14—H14	119.3
N8—C8—C7	113.7 (4)	O3—C16—H16A	110.3
C9—C8—N8	118.5 (4)	O3—C16—H16B	110.3
C9—C8—C7	127.7 (4)	O3—C16—C17	107.3 (7)
C8—C9—H9	117.8	H16A—C16—H16B	108.5
C8—C9—C10	124.5 (4)	C17—C16—H16A	110.3
C10—C9—H9	117.8	C17—C16—H16B	110.3
C11—C10—C9	120.9 (4)	C16—C17—H17A	109.5
C11—C10—C15	119.0 (5)	C16—C17—H17B	109.5
C15—C10—C9	120.1 (5)	C16—C17—H17C	109.5
C10—C11—S1	122.3 (4)	H17A—C17—H17B	109.5
C12—C11—S1	117.5 (4)	H17A—C17—H17C	109.5
C12—C11—C10	120.0 (5)	H17B—C17—H17C	109.5
C1—C2—H2	120.3		

### 2-(2-Bromo-5-fluorophenyl)-8-ethoxy-3-nitro-2H-thiochromene (A) Hydrogen-bond geometry (Å, º)

**Table:**

*D*—H···*A*	*D*—H	H···*A*	*D*···*A*	*D*—H···*A*
C9—H9···O2^i^	0.93	2.50	3.419 (7)	168

Symmetry code: (i) −*x*, −*y*+1, −*z*.

### 2-(2-Bromo-5-fluorophenyl)-7-methoxy-3-nitro-2H-thiochromene (B) Crystal data

**Table:**

C_16_H_11_BrFNO_3_S	*F*(000) = 792
*M**_r_* = 396.23	*D*_x_ = 1.749 Mg m^−^^3^
Monoclinic, *P*2_1_/*c*	Mo *K*α radiation, λ = 0.71073 Å
*a* = 7.6231 (4) Å	Cell parameters from 9945 reflections
*b* = 17.3484 (8) Å	θ = 3.0–28.3°
*c* = 11.8345 (6) Å	µ = 2.90 mm^−^^1^
β = 106.016 (2)°	*T* = 273 K
*V* = 1504.35 (13) Å^3^	Block, yellow
*Z* = 4	0.30 × 0.22 × 0.11 mm

### 2-(2-Bromo-5-fluorophenyl)-7-methoxy-3-nitro-2H-thiochromene (B) Data collection

**Table:**

Bruker D8 Quest CMOS diffractometer	3018 reflections with *I* > 2σ(*I*)
φ and ω scans	*R*_int_ = 0.032
Absorption correction: multi-scan (SADABS; Bruker, 2016)	θ_max_ = 28.4°, θ_min_ = 3.0°
*T*_min_ = 0.599, *T*_max_ = 0.746	*h* = −10→10
31660 measured reflections	*k* = −23→23
3745 independent reflections	*l* = −15→15

### 2-(2-Bromo-5-fluorophenyl)-7-methoxy-3-nitro-2H-thiochromene (B) Refinement

**Table:**

Refinement on *F*^2^	Primary atom site location: dual
Least-squares matrix: full	Hydrogen site location: inferred from neighbouring sites
*R*[*F*^2^ > 2σ(*F*^2^)] = 0.029	H-atom parameters constrained
*wR*(*F*^2^) = 0.070	*w* = 1/[σ^2^(*F*_o_^2^) + (0.0274*P*)^2^ + 0.9845*P*] where *P* = (*F*_o_^2^ + 2*F*_c_^2^)/3
*S* = 1.04	(Δ/σ)_max_ = 0.001
3745 reflections	Δρ_max_ = 0.52 e Å^−^^3^
209 parameters	Δρ_min_ = −0.49 e Å^−^^3^
0 restraints	

### 2-(2-Bromo-5-fluorophenyl)-7-methoxy-3-nitro-2H-thiochromene (B) Special details

**Table:**

Geometry. All esds (except the esd in the dihedral angle between two l.s. planes) are estimated using the full covariance matrix. The cell esds are taken into account individually in the estimation of esds in distances, angles and torsion angles; correlations between esds in cell parameters are only used when they are defined by crystal symmetry. An approximate (isotropic) treatment of cell esds is used for estimating esds involving l.s. planes.

### 2-(2-Bromo-5-fluorophenyl)-7-methoxy-3-nitro-2H-thiochromene (B) Fractional atomic coordinates and isotropic or equivalent isotropic displacement parameters (Å^2^)

**Table:**

	*x*	*y*	*z*	*U*_iso_*/*U*_eq_	
Br1	0.57090 (3)	0.36398 (2)	0.38157 (2)	0.04708 (9)	
S1	0.52686 (6)	0.31114 (3)	0.66185 (4)	0.03212 (11)	
F4	1.0275 (2)	0.57875 (8)	0.73645 (12)	0.0538 (4)	
O3	0.2529 (2)	0.42250 (10)	0.96347 (13)	0.0458 (4)	
O2	1.1678 (2)	0.23148 (10)	0.82659 (17)	0.0550 (4)	
O1	1.0798 (2)	0.26267 (11)	0.64265 (15)	0.0545 (4)	
N8	1.0577 (2)	0.26017 (10)	0.74190 (18)	0.0383 (4)	
C6	0.7901 (2)	0.40495 (10)	0.61148 (16)	0.0265 (4)	
C1	0.7106 (3)	0.43164 (11)	0.49777 (16)	0.0306 (4)	
C8	0.8921 (3)	0.29553 (11)	0.75735 (17)	0.0307 (4)	
C7	0.7581 (3)	0.32337 (11)	0.64724 (16)	0.0289 (4)	
H7	0.7692	0.2893	0.5835	0.035*	
C11	0.5550 (3)	0.34811 (11)	0.80401 (16)	0.0288 (4)	
C12	0.4044 (3)	0.37801 (11)	0.83133 (17)	0.0325 (4)	
H12	0.2966	0.3854	0.7721	0.039*	
C10	0.7218 (3)	0.33978 (11)	0.89180 (17)	0.0311 (4)	
C5	0.8972 (3)	0.45668 (11)	0.69165 (16)	0.0300 (4)	
H5	0.9539	0.4411	0.7682	0.036*	
C2	0.7313 (3)	0.50680 (13)	0.46588 (18)	0.0375 (5)	
H2	0.6742	0.5232	0.3898	0.045*	
C9	0.8784 (3)	0.30534 (11)	0.86622 (17)	0.0338 (4)	
H9	0.9746	0.2892	0.9287	0.041*	
C4	0.9184 (3)	0.53081 (12)	0.65674 (18)	0.0349 (4)	
C14	0.5759 (3)	0.39037 (13)	1.03475 (18)	0.0385 (5)	
H14	0.5829	0.4043	1.1118	0.046*	
C15	0.7271 (3)	0.36263 (12)	1.00596 (17)	0.0361 (4)	
H15	0.8368	0.3589	1.0646	0.043*	
C13	0.4121 (3)	0.39734 (12)	0.94672 (18)	0.0347 (4)	
C3	0.8361 (3)	0.55789 (12)	0.54603 (19)	0.0386 (5)	
H3	0.8503	0.6088	0.5256	0.046*	
C16	0.2464 (4)	0.43592 (16)	1.0811 (2)	0.0534 (6)	
H16A	0.1254	0.4515	1.0807	0.080*	
H16B	0.3313	0.4759	1.1157	0.080*	
H16C	0.2781	0.3894	1.1261	0.080*	

### 2-(2-Bromo-5-fluorophenyl)-7-methoxy-3-nitro-2H-thiochromene (B) Atomic displacement parameters (Å^2^)

**Table:**

	*U*^11^	*U*^22^	*U*^33^	*U*^12^	*U*^13^	*U*^23^
Br1	0.05237 (15)	0.05020 (14)	0.03012 (11)	−0.00306 (11)	−0.00293 (9)	−0.00516 (9)
S1	0.0285 (2)	0.0376 (3)	0.0274 (2)	−0.0057 (2)	0.00289 (18)	−0.00232 (19)
F4	0.0685 (9)	0.0376 (7)	0.0489 (8)	−0.0167 (7)	0.0057 (7)	−0.0092 (6)
O3	0.0422 (9)	0.0591 (10)	0.0385 (8)	0.0009 (7)	0.0153 (7)	−0.0034 (7)
O2	0.0376 (9)	0.0477 (10)	0.0733 (12)	0.0126 (7)	0.0044 (8)	0.0180 (9)
O1	0.0390 (9)	0.0724 (12)	0.0530 (10)	0.0034 (8)	0.0143 (8)	−0.0143 (9)
N8	0.0301 (9)	0.0276 (8)	0.0544 (11)	−0.0014 (7)	0.0066 (8)	−0.0011 (8)
C6	0.0260 (9)	0.0271 (9)	0.0274 (9)	0.0028 (7)	0.0091 (7)	0.0009 (7)
C1	0.0301 (9)	0.0346 (10)	0.0264 (9)	0.0027 (8)	0.0068 (7)	−0.0023 (8)
C8	0.0278 (9)	0.0244 (9)	0.0371 (10)	−0.0008 (7)	0.0044 (8)	0.0026 (8)
C7	0.0290 (9)	0.0277 (9)	0.0287 (9)	0.0002 (8)	0.0059 (7)	−0.0012 (7)
C11	0.0319 (10)	0.0275 (9)	0.0250 (9)	−0.0046 (7)	0.0046 (7)	0.0031 (7)
C12	0.0302 (9)	0.0353 (10)	0.0291 (9)	−0.0052 (8)	0.0032 (8)	0.0031 (8)
C10	0.0339 (10)	0.0275 (9)	0.0281 (9)	−0.0027 (8)	0.0019 (8)	0.0059 (7)
C5	0.0326 (10)	0.0312 (10)	0.0257 (9)	0.0012 (8)	0.0070 (7)	−0.0004 (8)
C2	0.0422 (11)	0.0393 (11)	0.0309 (10)	0.0086 (9)	0.0099 (9)	0.0083 (9)
C9	0.0318 (10)	0.0301 (10)	0.0341 (10)	−0.0011 (8)	0.0000 (8)	0.0073 (8)
C4	0.0378 (11)	0.0312 (10)	0.0362 (10)	−0.0032 (8)	0.0114 (8)	−0.0058 (8)
C14	0.0479 (12)	0.0390 (11)	0.0272 (10)	−0.0054 (10)	0.0081 (9)	−0.0003 (8)
C15	0.0381 (11)	0.0369 (11)	0.0273 (9)	−0.0032 (9)	−0.0010 (8)	0.0042 (8)
C13	0.0370 (11)	0.0341 (10)	0.0341 (10)	−0.0042 (9)	0.0115 (8)	0.0020 (8)
C3	0.0469 (12)	0.0295 (10)	0.0421 (11)	0.0029 (9)	0.0169 (10)	0.0051 (9)
C16	0.0579 (15)	0.0655 (16)	0.0437 (13)	−0.0029 (13)	0.0256 (12)	−0.0042 (12)

### 2-(2-Bromo-5-fluorophenyl)-7-methoxy-3-nitro-2H-thiochromene (B) Geometric parameters (Å, º)

**Table:**

Br1—C1	1.8961 (19)	C12—H12	0.9300
S1—C7	1.8307 (19)	C12—C13	1.392 (3)
S1—C11	1.7574 (19)	C10—C9	1.440 (3)
F4—C4	1.357 (2)	C10—C15	1.398 (3)
O3—C13	1.355 (3)	C5—H5	0.9300
O3—C16	1.426 (3)	C5—C4	1.374 (3)
O2—N8	1.221 (2)	C2—H2	0.9300
O1—N8	1.232 (2)	C2—C3	1.380 (3)
N8—C8	1.460 (3)	C9—H9	0.9300
C6—C1	1.394 (3)	C4—C3	1.370 (3)
C6—C7	1.516 (3)	C14—H14	0.9300
C6—C5	1.395 (3)	C14—C15	1.376 (3)
C1—C2	1.379 (3)	C14—C13	1.393 (3)
C8—C7	1.497 (3)	C15—H15	0.9300
C8—C9	1.332 (3)	C3—H3	0.9300
C7—H7	0.9800	C16—H16A	0.9600
C11—C12	1.377 (3)	C16—H16B	0.9600
C11—C10	1.410 (3)	C16—H16C	0.9600
			
C11—S1—C7	100.47 (9)	C4—C5—C6	119.55 (18)
C13—O3—C16	117.99 (18)	C4—C5—H5	120.2
O2—N8—O1	123.58 (19)	C1—C2—H2	119.8
O2—N8—C8	119.36 (19)	C1—C2—C3	120.50 (19)
O1—N8—C8	117.04 (17)	C3—C2—H2	119.8
C1—C6—C7	121.27 (17)	C8—C9—C10	123.18 (18)
C1—C6—C5	117.40 (17)	C8—C9—H9	118.4
C5—C6—C7	121.32 (16)	C10—C9—H9	118.4
C6—C1—Br1	120.14 (15)	F4—C4—C5	117.72 (18)
C2—C1—Br1	118.09 (15)	F4—C4—C3	119.08 (19)
C2—C1—C6	121.77 (18)	C3—C4—C5	123.20 (19)
N8—C8—C7	115.63 (17)	C15—C14—H14	120.5
C9—C8—N8	118.44 (18)	C15—C14—C13	118.98 (19)
C9—C8—C7	125.74 (18)	C13—C14—H14	120.5
S1—C7—H7	107.0	C10—C15—H15	118.8
C6—C7—S1	111.58 (13)	C14—C15—C10	122.39 (19)
C6—C7—H7	107.0	C14—C15—H15	118.8
C8—C7—S1	108.90 (13)	O3—C13—C12	115.12 (18)
C8—C7—C6	114.88 (15)	O3—C13—C14	124.95 (19)
C8—C7—H7	107.0	C12—C13—C14	119.93 (19)
C12—C11—S1	118.22 (14)	C2—C3—H3	121.2
C12—C11—C10	120.41 (18)	C4—C3—C2	117.55 (19)
C10—C11—S1	121.01 (15)	C4—C3—H3	121.2
C11—C12—H12	119.7	O3—C16—H16A	109.5
C11—C12—C13	120.61 (18)	O3—C16—H16B	109.5
C13—C12—H12	119.7	O3—C16—H16C	109.5
C11—C10—C9	121.27 (18)	H16A—C16—H16B	109.5
C15—C10—C11	117.59 (19)	H16A—C16—H16C	109.5
C15—C10—C9	121.07 (18)	H16B—C16—H16C	109.5
C6—C5—H5	120.2		

### 2-(2-Bromo-5-fluorophenyl)-7-methoxy-3-nitro-2H-thiochromene (B) Hydrogen-bond geometry (Å, º)

*Cg*3 is the centroid of the C10–C15 ring.

**Table:**

*D*—H···*A*	*D*—H	H···*A*	*D*···*A*	*D*—H···*A*
C9—H9···O1^i^	0.93	2.60	3.418 (3)	148
C15—H15···F4^ii^	0.93	2.54	3.268 (2)	136
C16—C16B···*Cg*3^iii^	0.96	2.86	3.668 (3)	143

Symmetry codes: (i) *x*, −*y*+1/2, *z*+1/2; (ii) −*x*+2, −*y*+1, −*z*+2; (iii) −*x*+1, −*y*+1, −*z*+2.

## References

[bb1] Bruker (2013). *APEX2* and *SAINT*. Bruker AXS Inc., Madison, Wisconsin, USA.

[bb2] Bruker (2016). *SADABS*. Bruker AXS Inc., Madison, Wisconsin, USA.

[bb3] Choudhury, A. R. & Mukherjee, S. (2013). *Adv. Synth. Catal.* **355**, 1989–1995.

[bb4] Dolomanov, O. V., Bourhis, L. J., Gildea, R. J., Howard, J. A. K. & Puschmann, H. (2009). *J. Appl. Cryst.* **42**, 339–341.

[bb5] Fouqué, A., Delalande, O., Jean, M., Castellano, R., Josselin, E., Malleter, M., Shoji, K. F., Hung, M. D., Rampanarivo, H., Collette, Y., van de Weghe, P. & Legembre, P. (2015). *J. Med. Chem.* **58**, 6559–6573. 10.1021/acs.jmedchem.5b00991 26237138

[bb6] Groom, C. R., Bruno, I. J., Lightfoot, M. P. & Ward, S. C. (2016). *Acta Cryst.* B**72**, 171–179. 10.1107/S2052520616003954 PMC4822653 27048719

[bb7] Horton, D. A., Bourne, G. T. & Smythe, M. L. (2003). *Chem. Rev.* **103**, 893–930. 10.1021/cr020033s 12630855

[bb8] Le, T. T. H., Youhei, C., Le, Q. H., Nguyen, T. B. & Mac, D. H. (2019). *Org. Biomol. Chem.* **17**, 6355–6358. 10.1039/c9ob01060b 31215939

[bb9] Nguyen, T. T. H., Nguyen, T. X., Cao, T. T. T., Dinh, T. H., Nguyen, H. H., Bui, T. T. T., Pham, V. P. & Mac, D. H. (2016). *Synlett*, **28**, 429–432.

[bb10] Sangeetha, S. & Sekar, G. (2017). *Org. Lett.* **19**, 1670–1673. 10.1021/acs.orglett.7b00462 28333469

[bb11] Sheldrick, G. M. (2015*a*). *Acta Cryst.* A**71**, 3–8.

[bb12] Sheldrick, G. M. (2015*b*). *Acta Cryst.* C**71**, 3–8.

[bb13] Simlandy, A. K. & Mukherjee, S. (2017). *J. Org. Chem.* **82**, 4851–4858. 10.1021/acs.joc.7b00579 28388053

